# Setting priorities for people with intellectual disability/intellectual developmental disorders across the lifespan: a call to action by the World Psychiatric Association

**DOI:** 10.1192/bji.2021.6

**Published:** 2021-08

**Authors:** Ashok Roy, Ken Courtenay, Mahesh Odiyoor, Patricia Walsh, Sarah Keane, Asit Biswas, Geoff Marston, Suchithra Thirulokachandran, Kerim Munir

**Affiliations:** 1 FRCPsych, Consultant Psychiatrist, Coventry and Warwickshire Partnership NHS Trust, UK. email: ashok.roy@covwarkpt.nhs.uk; 2 FRCPsych, Chair, Intellectual Disability Faculty, Royal College of Psychiatrists, London, UK; 3 FRCPsych, Strategic Clinical Director, Centre for Autism, Neurodevelopmental Disorders and Intellectual Disabilities, Cheshire and Wirral Partnership NHS Foundation Trust, UK; 4 MRCPsych, Chair of the Faculty of Learning Disability, College of Psychiatrists of Ireland, Ireland; 5 MRCPsych, Consultant Psychiatrist, HSE Dublin South Mental Health Services, Ireland; 6 FRCPsych, Consultant Psychiatrist, Leicestershire Partnership NHS Trust, UK; 7 FRCPsych, Consultant Psychiatrist, Coventry and Warwickshire Partnership NHS Trust, UK; 8 FRCPsych, Consultant Psychiatrist, Coventry and Warwickshire Partnership NHS Trust, UK; 9 DFAACAP, Director of Psychiatry, University Centre for Excellence in Developmental and Related Disabilities, Boston Children's Hospital, Harvard Medical School, Boston, Ma, USA

**Keywords:** Intellectual disability, international, policies, cultural differences, role for psychiatrists

## Abstract

People with DSM-5 intellectual disability/intellectual developmental disorder (ID/IDD) or ICD-11 disorders of intellectual development (DID) have multiple healthcare needs, but in many countries these needs are neither recognised nor managed effectively. This paper discusses the negative impact that stigma, discrimination and social exclusion have on the identification and care of persons with ID/IDD in low- and middle-income countries (LMICs). It also reviews different models of care for children, adolescents and adults. In discussing some initiatives in LMICs the emphasis is on early diagnosis, with success in providing locally sourced care for affected people and their families. This is where the medical, social and rights-based models of care intersect and is a premise of the person-centred biopsychosocial framework of the World Psychiatric Association's Presidential Action Plan 2020–2023. The plan invites psychiatrists to take a lead in changing the culture of care, as well as medical education, clinical training and research, with a renewed emphasis on workforce integration and service development in terms of community-based rehabilitation strategies.

Intellectual disability/intellectual developmental disorders (ID/IDD) in the American Psychiatric Association's DSM-5 or disorders of intellectual development (DID) in the World Health Organization's (WHO's) ICD-11 are currently considered under neurodevelopmental disorders. These international definitions of intellectual disability include the key criteria of significant impairment of intellectual and adaptive functioning arising before adulthood.

People with ID/IDD continue to face high risks in terms of social exclusion, stigma, discrimination and human rights violations across a multiplicity of low-resource settings, in particular in low- and middle-income countries (LMICs). Affected children and adolescents in particular lack early interventions and face difficulties in meeting developmental and educational milestones; many receive scarce assistance in managing developmental, social and environmental demands. Understanding of the global burden of disease (GBD), Mental Health Gap Action Programme (mhGAP) and disability-adjusted life-years (DALYs) metrics has yet to make an impact in implementation of policies and practices to improve quality of life, well-being, physical and mental health and rehabilitation in LMICs.

## Prevalence, identification and local perspectives

The changing terminology of ID/IDD has meant that it has been difficult to describe over the variations observed in its prevalence in surveys of the general population.^1^ The recognition of ID/IDD in the LMIC setting will depend on local factors, including expectations arising from educational provision and adaptive capacity. These influence the interpretation of current measures of intellectual and adaptive functioning. Identifying mild and moderate ID/IDD can also be challenging in contexts requiring rural outreach.^2^ Maulik et al^3^ estimated the global prevalence of ID/IDD to be approximately 1%, often correlated indirectly with national income. The prevalence of ID/IDD in LMICs is estimated to be almost double the level in high-income economies. In a systematic review, Fischer et al^4^ addressed the ascertainment issue and emphasised that locally developed screening tools can provide feasible measures of developmental disabilities in early life. They provided acceptable levels of sensitivity and specificity, had low cost and entailed only a short period of training in terms of health worker time for daily use. Identifying people with ID/IDD using population-based screening tools can be expensive and time consuming. Lakhan & Mawson^5^ showed a high correlation between ID/IDD identified in children in a formal survey conducted in an Indian tribal population and the proportion identified through focus group interviews in the communities where the children lived. They suggest that early engagement of communities in such surveys tends to accelerate acceptance of subsequent support. This entails openness, transparency and respect for cultural norms. A study of adults with ID/IDD in South Africa^6^ further illustrates the point that family carers are the gatekeepers and primary providers of the long-term care of people with ID/IDD. They expressed concerns about a lack of everyday external supports and education, training and life opportunities for their loved ones, which had the effect of preventing them developing their full potential.

In LMICs, children and youth with ID/IDD have traditionally had poor developmental outcomes compared to their unaffected peers, although with the important gains in terms of the United Nations Sustainable Development Goals, this is changing with greater recognition of the burden of ID/IDDs. Nevertheless, this has led to underfunding of ID/IDD services in terms of care priorities, which have also became increasingly protective and segregated, lacking a focus on vocational and life skill development. Masulani-Mwale et al^7^ describe a study in Malawi that aimed to reduce the psychological distress experienced by the parents of children with ID/IDD. They acknowledged the importance of taking into account the cultural context and taking a step-by-step approach in devising a non-pharmacological intervention for stressed families that was both feasible and acceptable to participants. The qualitative study provides a valuable insight into the lived experience of parents of children and youth with ID/IDD. The study demonstrated that culturally sensitive and effective interventions designed to reduce parental distress can be developed in LMIC settings, with important benefits to families. In the ultimate provision of evidence-based services in LMICs, there needs to be a convergence of service delivery models that acknowledge the way in which the local culture connotes disability and how persons with ID/IDD are marginalised in terms of stigma, sociocultural discrimination and environmental human rights constraints.^8^ Astbury & Walji^9^ highlight the additive effect of gender, disability and poverty (triple jeopardy). People with ID/IDD have increased contact with the criminal justice system, making up 7% of the adult prison population and a higher proportion in children and young people.^10^ A medical model of care is inappropriate and there is now a growing trend for practical and expert knowledge to be shared between families, who report having high expectations of what their children could achieve, given adequate support. Urgent restructuring of services and a change in the professional culture of educational, social and health organisations is needed to bridge the gulf between legislation and development of services for people with ID/IDD. Menon et al^11^ describe an initiative in India (the National Trust) that is developing group homes, respite services, caregiver training and promotion of community awareness based on a mixture of socioecological models of protective care, under a legal guardianship programme managed by local committees legally empowered to act in the best interests of individuals with ID/IDD. Although such a guardianship framework can be developed in other LMIC contexts, ultimately provision of community care for adults with ID/IDD is not only a financial challenge but needs to take into account cultural beliefs and values.

## The WPA Plan

The World Psychiatric Association (WPA) Presidential Action Plan 2020–2023 came into effect in October 2020. It sets important priorities that will affect the mental healthcare of people with ID/IDD in the hope of inspiring psychiatrists in our understanding of these complex neurodevelopmental disorders as an international priority. Given that globally, only a minority of persons with ID/IDD receive treatment for their disproportionately high experience of comorbid mental disorders,^1^ the need to support mental health professionals in primary and community-based healthcare settings and to engage with general healthcare workers remains paramount.

The Action Plan 2020–2023 seeks to organise activities in a global perspective targeting psychiatrists working in low-resource countries in order to meet the mental health needs of people with ID/IDD. The six areas of the WPA Action Plan include: (1) promotion of public mental health as a guiding principle; (2) promotion of child, adolescent and youth mental healthcare as both a preventive and secondary treatment strategy to alleviate future burden; (3) addressing physical and mental health related comorbidities especially in reference to the preponderance of non-communicable diseases across the lifespan that often go unrecognised in persons with ID/IDD; (4) building capacity in terms of undergraduate medical, postdoctoral education and training including opportunities for research; (5) partnering with other professional organisations and non-governmental organisations (NGOs); and (6) continuing the work of the WPA and its scientific sections and components within a collaborative framework, including its prior work on prevention of stigma related to mental illness.

The WPA Action Plan intends to develop guidelines that will apply to services that will emphasise the role to be played by psychiatrists in facilitating better care of persons with ID/IDD across the lifespan, working in partnership with nurses, therapists, psychologists, advocates and families. Psychiatrists and clinical psychologists often serve as arbiters of the enforcement of eligibility guidelines for service delivery for children, adolescents as well as adults across the lifespan. They are often called in to provide assessments and opinion in terms of guardianship and competency. The education of psychiatrists and all mental health professionals therefore can provide important opportunities for change. The members of the WPA Action Plan Working Group on ID/IDD are committed to collaborating with international and national agencies and parental self-help groups to promote awareness of and support for ID/IDD in order to reduce its considerable impact on society. The Working Group invites all stakeholders, professional societies, NGOs and governmental agencies in the health, education and social care sectors to collaborate in enhancing the well-being of persons with ID/IDD.

The barriers in care faced by persons with ID/IDD are reflected in varying degrees across many cultures irrespective of economic status. People with ID/IDD living in LMICs classified by the World Bank as low- and lower-middle-income economies face the greatest risk in restriction of social choices, rights, unattended care for physical and mental health related comorbidities, as well as increased mortality in the general population. In rural populations, between 72 and 94% of individuals with ID/IDD have one or more unmet health needs.^2^

## Models of disability

Persons with ID/IDD, in particular in LMICs, ought not to be considered at the margins of citizenship and human rights. Although ID/IDD is equated with limitation in competence, in many high-resource economies rights-based approaches have progressively been able to reclaim privileges and dignity. In contrast, in many LMICs, implementation of social inclusion models for persons with ID/IDD has faced cultural challenges, with stigma playing a major role in hindering acceptance. The missing link has been the limitation in regard and practice of human rights as a pathway toward greater advocacy for inclusive policies in LMICs.^12^

Currently, the participation of people with ID/IDD in LMICs in everyday activities is often inhibited by both cultural and human rights challenges that undermine opportunities for inclusion and in so doing do not acknowledge their abilities. The impact of stigma on families of persons with ID/IDD in LMICs is widespread and presents important challenges to the work ahead. Stigma is a highly complex sociocultural phenomenon residing in the holder of such attitudes and actions, and is not necessarily influenced by the nature of the disorder, age or gender of the person with ID/IDD, or the educational level of the caregiver. Accordingly, support for families must take into account their existing beliefs and support systems, including traditional cultural values as well as religious beliefs.

A social approach to disability founded on human rights and built upon cultural and societal norms is more likely to promote inclusion of people with ID/IDD in LMICs worldwide. Within such a cultural and social framework, communities need to be assisted in embracing disability, rather than rejecting it outright owing to stigma based on stereotypes, prejudice and discrimination. A human rights informed social approach also provides original and valuable insights into salient issues that psychiatrists and mental health professionals struggle with in their practice, since there are many historical, clinical and ethical parallels, as well as overlaps, between the plight of persons with ID/IDD and persons with severe mental illness. The WPA Presidential Action Plan will work within a global perspective focusing specifically on improving coverage of evidence-based interventions both to prevent and treat mental disorders and to promote mental well-being.

A change in values is essential, with an emphasis on person-centred attitudes, based on perceived strengths. An integrated approach, as in the biopsychosocial framework of the International Classification of Functioning, Disability and Health (ICF), developed by the WHO in parallel with ICD-11, captures health and disability at both the individual and the population levels, synthesising aspects of the medical model implicit in DSM-5 and ICD-11 with the social model without compromising the complexities inherent in the definition of disability. An important contribution is the consideration of the pervasive role played by the environment in contributing to heightening or reducing the impact of disability.

## Implications

The discussion on setting priorities in ID/IDD with a particular emphasis on LMICs will be considered in the light of a number of implications.

### Role of families

Notwithstanding significant cultural and financial obstacles and enduring the dismay of stigma and social exclusion of loved ones, parents and families of persons with ID/IDD in LMIC settings remain the primary providers of support throughout their lives. Integration of social and medical models is necessary to develop culturally acceptable community-based strategies in LMICs to address the needs of persons with ID/IDD and to support families at the local level, enabling persons with ID/IDD to access education and training as well as diagnostic and treatment services. In such a holistic framework human rights within LMICs is a key factor, including the premise of each individual's right to dignity, respect, autonomy and equality. People with ID/IDD ought to be free to form relationships and benefit from optimal social integration in terms of access to health, education and employment.

### Mental health of intellectual disability (MHID) services

The WPA Presidential Action Plan on ID/IDD emphasises a renewed focus on development of mental health services for persons with ID/IDD in LMICs. Globally, psychiatrists in LMICs will need to continue to engage in the care of persons with ID/IDD as well as their prevention, in particular contributing to early screening and identification of young people with developmental delay. As implicit in the DSM-5 and ICD-11 definitions, ID/IDD is a heterogeneous neurodevelopmental category that also coexists with other neurodevelopmental disorders, including autism and language, coordination and motor deficits, as well as other comorbid mental and physical conditions. Scientific study of ID/IDD in LMICs is an important mission that ought not to detract from comprehensive care of persons with ID/IDD in the community.

### Person-centred model

Individuals with ID/IDD need globally agreed effective models of prevention, care and treatment. A person-centred approach that integrates medical, social and rights-based perspectives in LMICs is akin to the normalisation principle. How psychiatrists contribute to the development of such conditions in LMICs will entail development of a trained workforce so that care becomes deliverable locally within established models of primary care. Such a decentralised approach will necessitate the integration of the medical and social models and adoption of person-centred care that treats each person with ID/IDD as an individual not merely defined by a disability. This requires embracing rights-based value systems to ensure that all citizens with ID/IDD and their carers get the equality and respect they deserve.

### Support by psychiatrists and training

Psychiatrists need to have experience in working with people with ID/IDD and mental health in ID/IDD should be included in their undergraduate and postgraduate curricula. They will need to provide ongoing support and education to allied health and social care professionals, who can ensure the availability of home-based support, training in activities of daily living (ADL) and provision of community-based rehabilitation (CBR), defined by the WHO as a community rehabilitation strategy focusing on equalisation of opportunities and social integration of persons with disabilities.

### Leadership from the WPA

Defining the level of input from high-income countries to LMICs will vary by country, programme and cultural context, but ultimately will need to be facilitated by each country's national health system, level of motivation for leadership in psychiatry to change, available resources and existing skilled personnel. The WPA Presidential Action Plan is poised to work with the WHO and other national and international societies, professional groups, NGOs and organisations representing people with ID/IDD to facilitate awareness, training and research collaborations to disseminate successful programmes and experiences.
